# Peretinoin, an Acyclic Retinoid, Inhibits Hepatitis B Virus Replication by Suppressing Sphingosine Metabolic Pathway In Vitro

**DOI:** 10.3390/ijms19020108

**Published:** 2018-01-23

**Authors:** Kazuhisa Murai, Takayoshi Shirasaki, Masao Honda, Ryogo Shimizu, Tetsuro Shimakami, Saki Nakasho, Natsumi Shirasaki, Hikari Okada, Yoshio Sakai, Taro Yamashita, Shuichi Kaneko

**Affiliations:** 1 Department of Gastroenterology, Kanazawa University Graduate School of Medical Science, Kanazawa 920-8641, Japan; k.murai.0612@gmail.com (K.M.); t-shirasaki@m-kanazawa.jp (T.S.); shimizuryo5@gmail.com (R.S.); shimakami@m-kanazawa.jp (T.S.); junwbfg0522@gmail.com (S.N.); takayoshi.shirasaki@gmail.com (N.S.); okada0922@gmail.com (H.O.); yoshios@m-kanazawa.jp (Y.S.); taroy@m-kanazawa.jp (T.Y.); skaneko@m-kanazawa.jp (S.K.); 2 Department of Advanced Medical Technology, Kanazawa University Graduate School of Health Medicine, Kanazawa 920-0942, Japan

**Keywords:** hepatitis B virus, acyclic retinoid, SPHK1, HDAC1

## Abstract

Hepatocellular carcinoma (HCC) frequently develops from hepatitis C virus (HCV) and hepatitis B virus (HBV) infection. We previously reported that peretinoin, an acyclic retinoid, inhibits HCV replication. This study aimed to examine the influence of peretinoin on the HBV lifecycle. HBV-DNA and covalently closed circular DNA (cccDNA) were evaluated by a qPCR method in HepG2.2.15 cells. Peretinoin significantly reduced the levels of intracellular HBV-DNA, nuclear cccDNA, and HBV transcript at a concentration that did not induce cytotoxicity. Conversely, other retinoids, such as 9-*cis*, 13-*cis* retinoic acid (RA), and all-*trans*-retinoic acid (ATRA), had no effect or rather increased HBV replication. Mechanistically, although peretinoin increased the expression of HBV-related transcription factors, as observed for other retinoids, peretinoin enhanced the binding of histone deacetylase 1 (HDAC1) to cccDNA in the nucleus and negatively regulated HBV transcription. Moreover, peretinoin significantly inhibited the expression of SPHK1, a potential inhibitor of HDAC activity, and might be involved in hepatic inflammation, fibrosis, and HCC. SPHK1 overexpression in cells cancelled the inhibition of HBV replication induced by peretinoin. This indicates that peretinoin activates HDAC1 and thereby suppresses HBV replication by inhibiting the sphingosine metabolic pathway. Therefore, peretinoin may be a novel therapeutic agent for HBV replication and chemoprevention against HCC.

## 1. Introduction

Hepatitis B virus (HBV) infection is the major cause of hepatocellular carcinoma (HCC), and 350 million people worldwide are chronic carriers of HBV [1]. HBV is a DNA virus and has a 3.2 kb-long relaxed circular genome with overlapping open reading frames. Nucleos(t)ide analog (NA) therapy for chronic hepatitis B patients has been effective in suppressing HBV replication; however, NA could not completely eliminate HBV covalently closed circular DNA (cccDNA) in the nucleus [2]. Therefore, novel antiviral agents targeting cccDNA are required to cure HBV infection. 

Peretinoin (generic name; code, NIK-333) is a novel synthetic acyclic retinoid (peretinoin) that was developed by Kowa company, Ltd. (Aichi, Japan). Peretinoin is an oral acyclic retinoid with a vitamin A-like structure that targets retinoid nuclear receptors [3]. The administration of peretinoin significantly reduced the incidence of post-therapeutic HCC recurrence and improved the survival rates of patients in a clinical trial [4]. Larger-scale clinical trials are now ongoing in various countries to confirm its clinical efficacy in preventing HCC associated with HBV and HCV after curative treatment. We reported that peretinoin prevented the development of hepatic fibrosis and tumors using a platelet-derived growth factor (PDGF)-C transgenic mouse model [5]. We also reported that peretinoin inhibited HCV replication and virus release by altering intracellular lipid metabolism [6]. However, the importance of the peretinoin in HBV replication remains poorly understood. 

Recently, we reported that peretinoin inhibited hepatocarcinogenesis by suppressing sphingosine kinase 1 (SPHK1) expression and its activity [7]. SPHKs (SPHK1 and SPHK2) and their bioactive product, sphingosine-1-phosphate (S1P), are important regulators of inflammation and many cancers [8]. S1P regulates many cellular processes, including cell growth, cytokine and chemokine production, and cell survival and invasion [9]. Moreover, S1P specifically binds to the histone deacetylases HDAC1 and HDAC2 and inhibits their enzymatic activity [10]. HBV cccDNA has two major forms of epigenetic regulation [11]. The hyperacetylation of cccDNA-bound histones is associated with increased HBV replication. On the other hand, the hypoacetylation of cccDNA-bound H3 and H4 histones and acetyltransferases p300/CREB-binding protein (CBP) and HDAC1 is associated with low HBV replication [12]. 

In this study, we evaluated the effects of peretinoin on HBV replication. We demonstrate that peretinoin inhibits HBV replication by reducing the mRNA and protein levels of SPHK1 and its transcription factor, Egr-1, which leads to the recruitment of HDAC1 to cccDNA. 

## 2. Results

### 2.1. Peretinoin Inhibits HBV Replication

**Figure 1:**
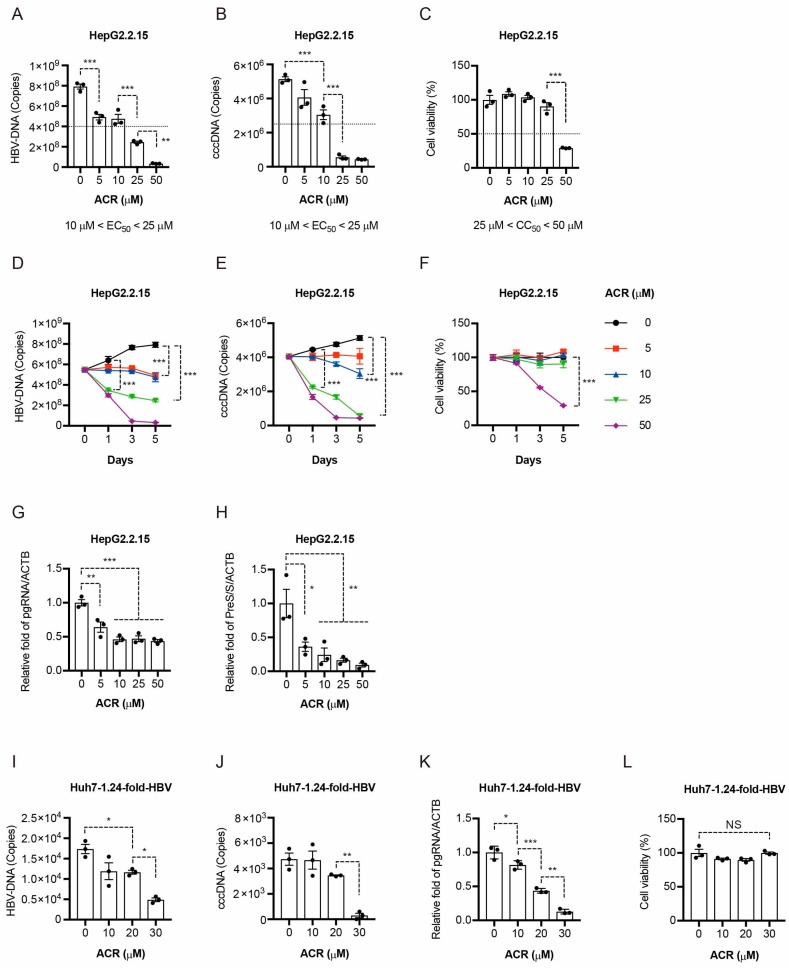
Peretinoin inhibits HBV replication. (**A**–**H**) HepG2.2.15 cells were treated with 0, 5, 10, 25, or 50 μM of peretinoin for 5 days. (**A**) HBV-DNA levels were analyzed by qPCR. (**B**) cccDNA levels were analyzed by qPCR. (**C**) Cytotoxicity was analyzed by MTT assay after the inoculation of peretinoin for 5 days. (**D**) Time-dependent effect of peretinoin on HBV-DNA. (**E**) Time-dependent effect of peretinoin on cccDNA. (**F**) Time-dependent effect of peretinoin on cytotoxicity. (**G**) Pregenomic RNA levels of HBV were analyzed by RTD-PCR, and pregenomic RNA levels were normalized against the β-actin mRNA level. (**H**) PreS/S RNA levels were analyzed by RTD-PCR, and PreS/S RNA levels were normalized against the β-actin mRNA level. (**I**–**L**) A plasmid encoding 1.24-fold-HBV was transfected into Huh7 cells and 24 h after transfection, 0, 10, 20, or 30 μM of peretinoin was added for 5 days. (**I**) HBV-DNA levels were analyzed by qPCR. (**J**) cccDNA levels were analyzed by qPCR. (**K**) Pregenomic RNA levels of HBV were analyzed by RTD-PCR. (**L**) Cytotoxicity was analyzed by MTT assay after the inoculation of peretinoin for 5 days. The data are represented as means ± SEM from three independent experiments. * *p* < 0.05, ** *p* < 0.01, *** *p* < 0.001.

Peretinoin inhibits HCV replication [6]. To examine whether peretinoin exhibits anti-HBV activity, we treated HepG2.2.15 cells, which have been stably transformed with HBV genome in HepG2 cells, with peretinoin in a dose-dependent manner. Five days after peretinoin treatment, qPCR analysis was performed to determine HBV-DNA and cccDNA. Peretinoin repressed cellular HBV-DNA levels in a dose-dependent manner (Figure 1A). Interestingly, peretinoin also repressed nuclear cccDNA levels in a dose-dependent manner (Figure 1B). The range of antiviral half maximal effective concentrations (EC_50_) of peretinoin was 10–25 μM (Figure 1A,B). We also determined the half cytotoxicity concentrations (CC_50_) of peretinoin in HepG2.2.15 cells using 3-(4,5-dimethylthiazolyl-2)-2,5-diphenyltetrazolium bromide (MTT) assay. The range of CC_50_ of peretinoin was 25–50 μM (Figure 1C). The time-dependent effect on HBV replication showed that peretinoin concentrations of 25 and 50 μM inhibited HBV replication from the start of the experiment (Day 1; Figure 1D,E), with no cellular cytotoxicity observed at this point (Figure 1F). An enzyme-linked immunosorbent assay (ELISA) analysis of HBeAg in the culture medium at Day 3 showed a significant reduction in its levels, by about 50%, compared with the control. Next, we examined the effect of peretinoin on the pregenomic RNA and PreS/S RNA transcription using RTD-PCR. Peretinoin repressed the expression of pregenomic RNA and PreS/S RNA in HepG2.2.15 cells (Figure 1G,H). The findings were also confirmed by other hepatoma cell lines, Huh7 cells. Huh7 cells were transfected with 1.24-fold-HBV plasmid. Then, the transfected cells were treated with peretinoin. Peretinoin strongly repressed the levels of HBV-DNA (Figure 1I), cccDNA (Figure 1J), and pregenomic RNA (Figure 1K) without cytotoxicity (Figure 1L). These results suggest that peretinoin exhibited anti-HBV activity at effective antiviral concentrations that did not induce cytotoxicity.

### 2.2. Other Retinoids Do Not Exhibit Anti-HBV Activity

**Figure 2:**
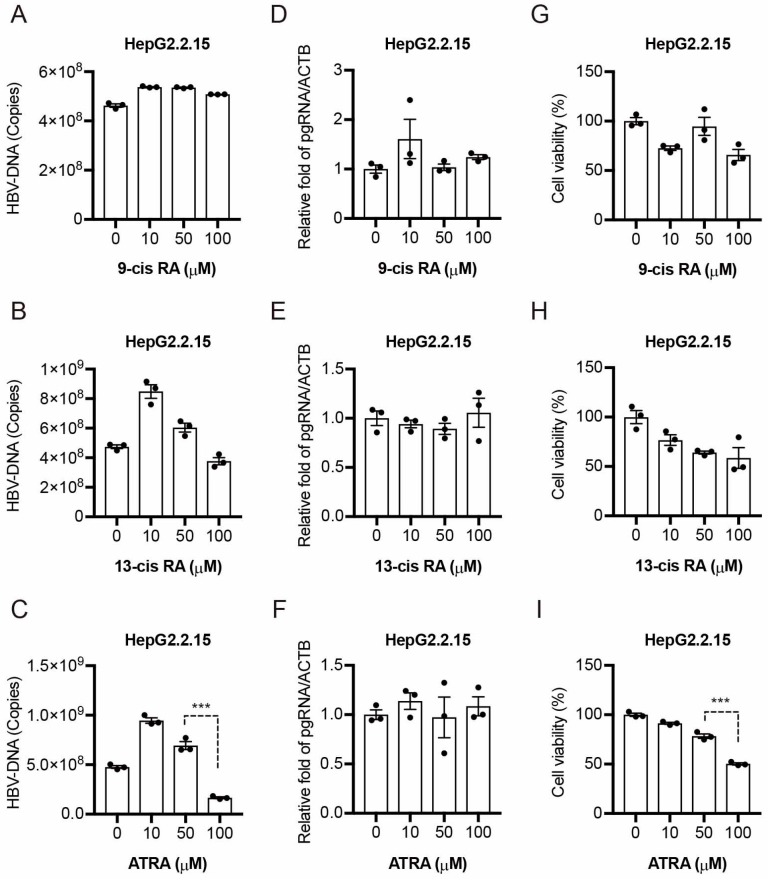
Other retinoids do not exhibit anti-HBV activity. HepG2.2.15 cells were treated with ATRA, 9-*cis* RA, and 13-*cis* RA at a concentration of 10, 50, and 100 μM, respectively, for 5 days. (**A**–**C**) HBV-DNA levels were analyzed by qPCR. (**D**–**F**) Pregenomic RNA levels of HBV were analyzed by RTD-PCR, and pregenomic RNA levels were normalized against the β-actin mRNA level. (**G**–**I**) Cytotoxicity was analyzed by MTT assay after the inoculation of retinoids for 5 days. The data are represented as means ± SEM from three independent experiments. *** *p* < 0.001.

We examined the effects of three different retinoids: all-*trans*-retinoic acid (ATRA), 9-*cis*-retinoic acid (9-*cis* RA), and 13-*cis*-retinoic acid (13-*cis* RA), on HBV replication. Because peretinoin repressed HBV replication at a 10 μM concentration, we treated HepG2.2.15 cells with ATRA, 9-*cis* RA, and 13-*cis* RA at a concentration of 10, 50, and 100 μM, respectively. Five days post-treatment, we evaluated the levels of HBV-DNA, the expression of pregenomic RNA, and cell viability. Interestingly, 9-*cis* RA and 13-*cis* RA did not repress HBV-DNA (Figure 2A,B) and pregenomic RNA (Figure 2D,E) within the range of no cytotoxicity (Figure 2G,H). ATRA repressed HBV-DNA at a 100 μM concentration (Figure 2C). However, the concentration showed significant cytotoxicity (Figure 2I). 

Contrarily, we found that peretinoin specifically repressed HBV replication at a concentration of 10 μM without inducing cytotoxicity (Figure 1A–F). These results suggested that peretinoin uniquely repressed HBV replication.

### 2.3. Peretinoin Inhibits the Egr-1-SPHK1 Axis in HBV-Replicating Cells

**Figure 3:**
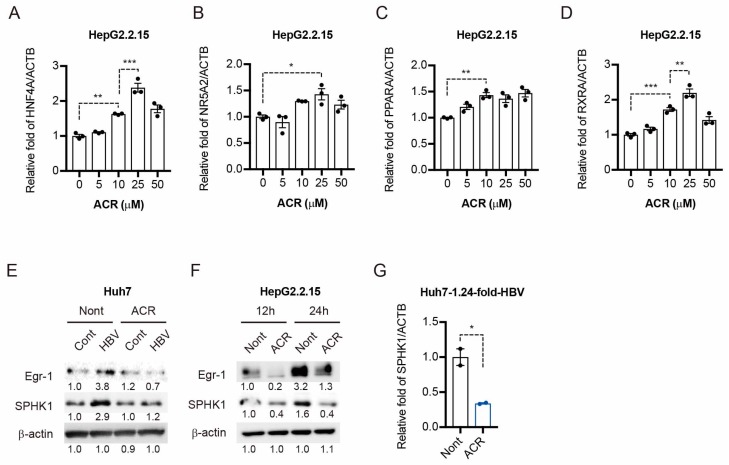
Peretinoin inhibits Egr-1-SPHK1 axis in HBV-replicating cells. (**A**–**D**) HepG2.2.15 cells were treated with 0, 5, 10, 25, or 50 μM of peretinoin for 5 days. qRT-PCR analysis of HNF4A (**A**), NR5A2 (**B**), PPARα (**C**), and RXRα (**D**) mRNA in peretinoin-treated HepG2.2.15 cells. Results were normalized to those of the β-actin mRNA level. (**E**,**G**) A plasmid encoding 1.24-fold-HBV or a control empty vector was transfected into Huh7 cells and 24 h after transfection, 30 μM of peretinoin was added for 3 days. Egr-1, SPHK1, and β-actin protein levels were detected by Western blotting analysis in Huh7 cells (**E**) and qRT-PCR analysis of SPHK1 mRNA in peretinoin-treated HepG2.2.15 cells (**G**). (**F**) HepG2.2.15 cells were treated with peretinoin at a concentration of 0 or 30 μM. 12 h and 24 h after inoculation, Egr-1, SPHK1, and β-actin protein levels were determined by Western blotting analysis (**F**). The data are represented as means ± SEM. * *p* < 0.05, ** *p* < 0.01, *** *p* < 0.001.

Hepatocyte nuclear factor 4 (HNF4), nuclear receptor subfamily 5 group A member 2 (NR5A2), peroxisome proliferator-activated receptor alpha (PPARα), and retinoid X receptor alpha (RXRα) were representative transcriptional factors activating the HBV transcription [13]. To explore the possibility that the downregulation of these transcription factors by peretinoin might be the reasons for the suppression of HBV replication, we measured these genes’ expression in HepG2.2.15 cells. Interestingly, the results of RTD-PCR analysis showed that HNF4A (Figure 3A), NR5A2 (Figure 3B), PPARα (Figure 3C), and RXRα (Figure 3D) mRNA levels were slightly induced by peretinoin. Therefore, the suppression of HBV replication by the peretinoin was not due to the changes in these transcription factors. S1P is a sphingolipid metabolite and specifically binds to the HDAC1 and inhibits this enzymatic activity [10]. S1P is formed by two closely related sphingosine kinases, SPHK1 and SPHK2. The early growth response protein 1 (Egr-1) is one of the SPHK1 transcription factors [14]. We first examined whether HBV replication could induce Egr-1 and SPHK1 expression. Interestingly, Western blotting analysis clearly showed that Egr-1 and SPHK1 protein levels were induced in HBV-replicating cells (Figure 3E). Interestingly, peretinoin suppressed HBV-induced upregulation of Egr-1 and SPHK1 expression in both mRNA and protein levels (Figure 3F,G). These results suggest that peretinoin strongly suppresses Egr-1-SPHK1 expression induced by HBV replication.

### 2.4. Peretinoin Enhances HDAC1-cccDNA Binding Activity via Suppression of SPHK1 Activity

**Figure 4:**
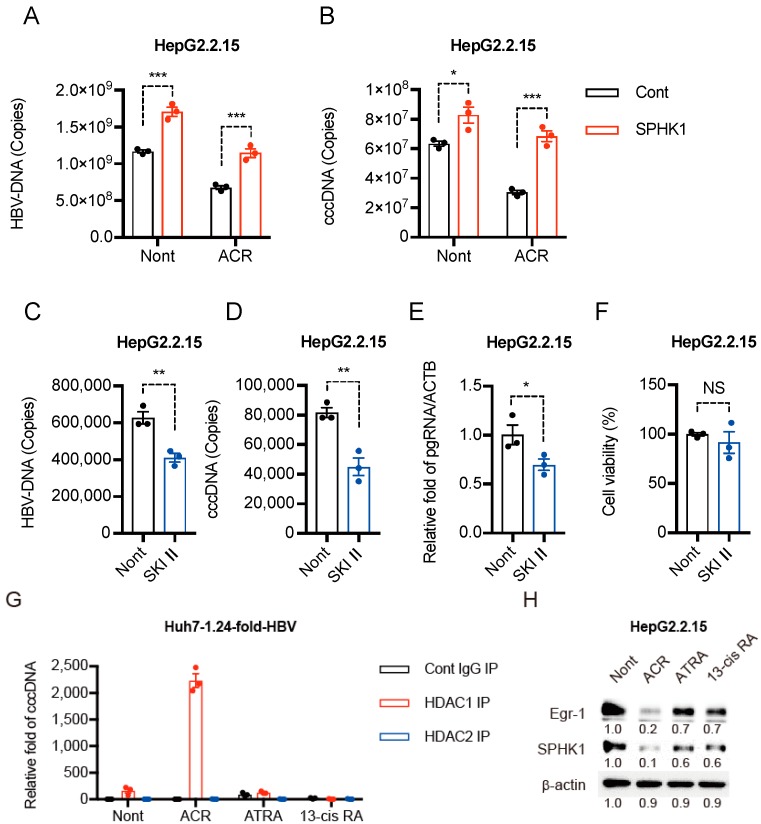
Peretinoin enhances HDAC1-cccDNA binding activity via the suppression of SPHK1 activity. (**A**,**B**) A plasmid encoding SPHK1 or a control empty vector was transfected into HepG2.2.15 cells and 24 h after transfection, 0 or 30 μM of peretinoin was added for 5 days. (**A**) HBV-DNA levels were analyzed by qPCR. (**B**) cccDNA levels were analyzed by qPCR. (**C**–**F**) HepG2.2.15 cells were treated with 0 or 10 μM of SKI II for 3 days. (**C**) HBV-DNA levels were analyzed by qPCR. (**D**) cccDNA levels were analyzed by qPCR. (**E**) Pregenomic RNA levels of HBV were analyzed by RTD-PCR, and pregenomic RNA levels were normalized against the β-actin mRNA level. (**F**) Cytotoxicity was analyzed by MTT assay. (**G**) Huh7 cells were transfected with 1.24-fold-HBV plasmid. Twenty-four hours after transfection, peretinoin, ATRA, or 13-*cis* RA was added for 3 days. Chromatin was immunoprecipitated with the control IgG or anti-HDAC1 antibodies and analyzed by qPCR with HBV cccDNA selective primers. (**H**) HepG2.2.15 cells were treated with 30 μM of peretinoin, ATRA, or 13-*cis* RA. Twenty-four hours after inoculation, Egr-1, SPHK1, and β-actin protein levels were determined by Western blotting analysis. The data are represented as means ± SEM from three independent experiments. * *p* < 0.05, ** *p* < 0.01, *** *p* < 0.001.

We recently reported that peretinoin reduced the mRNA, protein levels, and enzymatic activity of SPHK1 [7]. We examined whether the overexpression of SPHK1 could compensate for the suppression of HBV replication induced by peretinoin. When SPHK1 was overexpressed in HepG2.2.15 cells before peretinoin treatment, the suppressive effect of peretinoin on HBV replication was cancelled (Figure 4A,B). Next, we examined whether an SPHK1 inhibitor, SKI II, could inhibit HBV replication in HepG2.2.15 cells. As expected, SKI II repressed the levels of HBV-DNA (Figure 4C), cccDNA (Figure 4D), and the expression of pregenomic RNA (Figure 4E), although no significant cytotoxicity was observed (Figure 4F). These results suggest that the SPHK1-S1P pathway might be beneficial for the HBV lifecycle. Finally, to assess whether HDACs interact with the cccDNA, we performed an anti-HDACs cccDNA Chip assay in HBV replicating Huh7 cells. We found that HDAC1 strongly binds to the cccDNA in the presence of peretinoin but not ATRA or 13-*cis* RA (Figure 4G). We also found that peretinoin strongly reduced the levels of Egr-1 and SPHK1 protein more than ATRA or 13-*cis* RA in HepG2.2.15 cells (Figure 4H). These results suggest that peretinoin strongly and specifically suppressed SPHK1 activity, leading to HDAC1 recruitment to cccDNA. 

## 3. Discussion

This study demonstrated for the first time that peretinoin inhibited HBV replication using two hepatoma cell lines: HepG2.2.15 cells, which have been stably transformed with HBV genome into HepG2 cells, and Huh7 cells. We clearly showed that peretinoin suppressed HBV replication in a dose-dependent manner within the range of peretinoin showing no cytotoxicity (Figure 1). Interestingly, the anti-HBV effect was peretinoin specific, as we examined the effects of three different retinoids, ATRA, 9-*cis* RA, and 13-*cis* RA, on HBV replication and we observed no anti-HBV effect of these retinoids. Because current treatment for the patients with CHB using nucleos(t)ide analog (NA) does not eliminate the viral cccDNA [15], the combination of peretinoin and NA might be an alternative therapy for HBV infection. Further research should be performed to validate the anti-HBV effect by peretinoin using human primary hepatocytes.

Contrary to the results we observed with peretinoin, a previous report showed that ATRA and 9-*cis* RA increased HBV replication [16,17]. We therefore hypothesized that the antiviral effect of peretinoin against HBV would be related to the expression of transcription factors that stimulate HBV transcription. Unexpectedly, many transcription factors (HNF4A, NR5A2, PPARα, and RXRα) that could activate the HBV promoter were upregulated in peretinoin-treated HepG2.2.15 cells (Figure 3A–D), indicating that peretinoin enhanced rather than suppressed the expression of HBV transcription factors. Therefore, the expression levels of HBV transcription factors were irrelevant to the anti-HBV effect of peretinoin. 

The HBV cccDNA accumulation in minichromosomes is organized by histone, non-histone viral protein (HBx), and cellular proteins. In high viral replication of HBV, cccDNA-associated histones are hyperacetylated and pregenomic RNA is actively transcribed. Pollicino et al. clearly showed that a low HBV replication status correlated with cccDNA hypoacetylation and recruitment of HDAC1 [18]. Belloni et al. also confirmed this phenomenon using HBx mutant models. In cells replicating HBx mutant HBV, the recruitment of the histone deacetylases hSirtl and HDAC1 onto cccDNA was increased, and HBV replication was then suppressed [19]. On the other hand, recent genome-wide mapping of histone acetyltransferase (HAT) and HDAC binding to chromatin showed that both were targeted to the transcribed regions of active genes by phosphorylated RNA Pol II. The majority of HDACs in the human genome function to reset chromatin by removing the acetylation of the active genes and maintaining the acetylation balance of the human genome [20].

We recently reported that peretinoin significantly suppressed SPHK1 mRNA and protein levels, and its bioactive product, S1P [7]. We showed that SPHK1 contributed to the hepatocarcinogenesis in DEN-induced mouse hepatoma models using SPHK1 knockout mice [7]. Moreover, recent reports accumulated the significance of SPHK–S1P axis for hepatic inflammation and fibrosis [21,22]. In addition, Hait et al. reported that S1P inhibited the activity of HDAC1 and HDAC2 that formed repressor complexes on the promoter region [10]. These reports led us to hypothesize that the repression of HBV replication by peretinoin could be due to the epigenetic modifications of cccDNA. In the present study, we found that the expression of SPHK1 protein was induced in HBV-replicating cells (Figure 3E,F). Interestingly, the expression of Egr-1, a transcription factor for the positive regulation of SPHK1 [14], was also induced in HBV-replicating cells. Importantly, we showed that peretinoin strongly reduced Egr-1 and SPHK1 in protein levels (Figure 3E,F). It was also reported that HBx proteins induced SPHK1 [23]. We and other groups showed the involvement of Egr-1 for hepatic fibrosis [24], hepatocarcinogenesis [25], tumor malignancy [26], and chemo resistance [27]. These results suggested that HBV infection enhanced Egr-1-SPHK1-S1P axis.

These collective results caused us to speculate that in HBV-replicating cells, HAT and HDAC repressor complexes, including SPHK1, could be recruited to the active transcription site of the cccDNA. However, SPHK1, whose expression is induced by HBV infection, inhibits HDAC activity, so active HBV replication continues. The peretinoin-induced inhibition of SPHK1 restored HDAC activity, and the HDAC was then able to deacetylate the cccDNA. The phenotype induced by peretinoin might be more obvious with cccDNA than cellular DNA because of the more active transcription of cccDNA. In this study, we showed that HDAC1 strongly bound to the cccDNA in the presence of peretinoin, which was demonstrated using the cccDNA Chip assay with an anti-HDAC antibody (Figure 4G). Our results also showed that the suppressive effect of HDAC on HBV transcription might be exerted over the activating effect of the HBV transcriptional factors. This is probably because the deacetylation caused by HDAC could change the structure of the cccDNA from an open to a closed form, and such cccDNA structural changes may prevent the HBV transcriptional factors from accessing the cccDNA. Further studies should be performed to clarify these findings.

In this study, we could not clarify how the effects of these retinoids were different. However, recent reports showed peretinoin induces nuclear localization of transglutaminase 2 [28] that induces Sp1 cross-linking and inactivation [29]. It is reported that Sp1 is involved in the positive regulation for the expression of both Egr-1 [30] and SPHK1 mRNA [7]. Further research should be performed to clarify the differences in the molecular aspects of peretinoin and other retinoids. Collectively, our results indicate that peretinoin activates HDAC1-related cccDNA modification by inhibiting the Egr-1-SPHK1-S1P axis, thereby suppressing HBV replication and potentially further repressing inflammation, fibrosis, and hepatocarcinogenesis of CHB.

## 4. Materials and Methods

### 4.1. Cells

HepG2.2.15 cells and Huh7 cells were maintained in DMEM (Thermo Fisher Scientific, Waltham, MA, USA) supplemented with 10% fetal bovine serum (Thermo Fisher Scientific), 1% l-glutamine (Thermo Fisher Scientific), and 1% penicillin/streptomycin (Thermo Fisher Scientific) in a humidified atmosphere of 5% CO_2_ at 37 °C.

### 4.2. Reagents

Acyclic retinoid (peretinoin) was kindly provided by Kowa Company (Aichi, Japan). All-trans-retinoic acid (ATRA), 9-*cis*-retinoic acid (9-*cis* RA), and 13-*cis*-retinoic acid (13-*cis* RA) were purchased from Sigma-Aldrich (St. Louis, MO, USA). Sphingosine kinase inhibitor 2 (SKI II, formally 4-[[4-(4-chlorophenyl)-2-thiazolyl]amino]-phenol) was purchased from Cayman Chemical Company (CAS No 312636-16-1; Ann Arbor, MI, USA).

### 4.3. Plasmids

The 1.24-fold-HBV/C plasmid was kindly provided by Dr. Yasuhito Tanaka, Nagoya City University [31]. A mammalian expression plasmid of N-terminal myc-FLAG-tagged human SPHK1 (GenBank data base accession number NM_021972) was purchased from OriGene (Rockville, MD, USA). 

### 4.4. Transfection

Plasmid transfection was performed according to the manufacturer’s instructions using Lipofectamine 3000 Regent (Thermo Fisher Scientific).

### 4.5. Quantification of HBV-DNA and cccDNA by qPCR

HBV-DNA was extracted from cells using a DNeasy Blood & Tissue Kit (Qiagen, Hilden, Germany) according to the manufacturer’s instructions. HBV-DNA was quantified by qPCR analysis as previously described [32]. The extracted DNA (50 ng) were treated for 60 min at 37 °C with 10U Plasmid safe DNase I (Epicentre, Madison, WI, USA) and then for 30 min at 70 °C for DNase inactivation. cccDNA (2.5 ng DNA) was quantified by qPCR analysis as previously described [32].

### 4.6. Quantitative RTD-PCR

Total RNA was isolated using the GenEluteTM Mammalian Total RNA Miniprep Kit (Sigma-Aldrich Japan K.K., Tokyo, Japan), and cDNA was synthesized using the High Capacity cDNA reverse transcription kit (Applied Biosystems, Carlsbad, CA, USA). RTD-PCR was performed using the 7500 Real Time PCR System (Applied Biosystems, Carlsbad, CA, USA) according to the manufacturer’s instructions. The primer pairs and probes were 5′-GCTCTGTATCGGGAGGCCTTA-3′ and 5′-TGAGTGCTGTATGGTGAGGAGAA-3′ as primer and 5′-FAM-AGTCTCCGGAACATT-MGB-3′ as probes for pregenomic RNA, and 5′-ACCCCAACAAGGATCATTGG-3′ and 5′-CGAATGCTCCCGCTCCTA-3′ as primer and 5′-FAM-CAGAGGCAAATCAG-MGB-3′ as probes for PreS/S, respectively. The primer pairs and probes for HNF4A, NR5A2, PPARα, RXRα, SPHK1, and ACTB were obtained from the TaqMan assay reagents library. RTD-PCR was performed using the 7500 Real Time PCR System (Applied Biosystems, Carlsbad, CA, USA) according to the manufacturer’s instructions. 

### 4.7. Western Blotting

Western blotting was performed as previously described [32]. Cells were washed in phosphate buffered saline (PBS) and lysed in radioimmunoprecipitation assay (RIPA) buffer containing complete Protease Inhibitor Cocktail and PhosSTOP (Roche Applied Science, Indianapolis, IN, USA). Membranes were blocked in Blocking One solution (Nacalai Tesque, Kyoto, Japan), and the expression of Egr-1, SPHK1, and β-actin was evaluated with a rabbit anti-EGR1 antibody, rabbit anti-SPHK1 antibody, and rabbit anti-β-actin antibody (Cell Signaling Technology Inc., Danvers, MA, USA), respectively.

### 4.8. Cytotoxicity Assay

HepG2.2.15 cells and HBV plasmid transfected Huh7 cells were seeded in 12-well plates at a density of 5 × 10^5^ cells per well and treated with retinoids at the indicated concentrations in the absence or presence of ETV or SKI II. Five days after treatment, the cell viability was determined using the Cell Counting Kit-8 (DOJINDO, Kumamoto, Japan) according to the manufacturer’s instructions.

### 4.9. cccDNA ChIp Assay

A cccDNA ChIP assay was carried out using a ChIP IT Express Enzymatic Kit (Active Motif, Carlsbad, CA, USA) according to the manufacturer’s instructions. Cells were treated with retinoids for 24 h before being fixed and homogenized. Following centrifugation, the supernatant was used for chromatin samples. Chromatin samples were incubated with a ChIP-grade anti-HDAC1 and HDAC2 antibody (Cell Signaling) overnight at 4 °C. Following washing and elution, DNA samples were treated for 60 min at 37 °C with 10U Plasmid safe DNase I and then for 30 min at 70 °C for DNase inactivation. cccDNA (2.5 ng DNA) was quantified by qPCR analysis as previously described.

### 4.10. Statistics

The results are expressed as the mean ± standard error (SEM). Significance was defined as *p* < 0.05 and was tested using the Student’s *t*-test or the paired *t*-test. Statistical analyses were performed using GraphPad Prism 7 (La Jolla, CA, USA).

## 5. Conclusions

Peretinoin activates HDAC1-related cccDNA modification by inhibiting the Egr-1-SPHK1-S1P axis, thereby suppressing HBV replication and potentially further repressing inflammation, fibrosis, and hepatocarcinogenesis of CHB. 

## Abbreviations

HBV	hepatitis B virus
cccDNA	covalently closed circular DNA
CHB	chronic hepatitis B
HCC	hepatocellular carcinoma
ACR	acyclic retinoid
SPHK	sphingosine kinase
S1P	sphingosine-1-phosphate
EGR-1	early growth response protein 1
HDAC	histone deacetylase
